# Extracellular redox sensitivity of Kv1.2 potassium channels

**DOI:** 10.1038/s41598-017-08718-z

**Published:** 2017-08-22

**Authors:** Victoria A. Baronas, Runying Y. Yang, Harley T. Kurata

**Affiliations:** Department of Pharmacology, Alberta Diabetes Institute, University of Alberta, Edmonton, Alberta Canada

## Abstract

Kv1.2 is a prominent potassium channel subtype in the nervous system and serves as an important structural template for investigation of ion channel function. However, Kv1.2 voltage-dependence exhibits dramatic cell-to-cell variability due to a gating mode shift that is regulated by an unknown mechanism. We report that this variable behavior is regulated by the extracellular redox environment. Exposure to reducing agents promotes a shift in gating properties towards an ‘inhibited’ gating mode that resists opening, and causes channels to exhibit pronounced use-dependent activation during trains of repetitive depolarizations. This sensitivity to extracellular redox potential is absent in other Kv1 channels, but is apparent in heteromeric channels containing Kv1.2 subunits, and overlaps with the reported physiological range of extracellular redox couples. Mutagenesis of candidate cysteine residues fails to abolish redox sensitivity. Therefore, we suggest that an extrinsic, redox-sensitive binding partner imparts these properties.

## Introduction

Kv1.2 is a voltage-gated potassium channel subtype that is most prominently expressed in the central nervous system, where it assembles with other members of the Kv1 channel subfamily, auxiliary β-subunits, and potentially other interacting partners that may regulate expression and function^1–3^. Kv1.2 has become a valuable model for investigation of ion channel gating mechanisms, as it was the first eukaryotic voltage-gated ion channel for which an atomic resolution structure was reported^4^. Thus, it has served as an essential structural template for the interpretation of functional data in a variety of ion channel types, and for the generation and simulation of *in silico* models of ion channels. Despite the understanding implied by the precision of an atomic resolution structure, there is remarkable variability of Kv1.2 gating behavior in different experimental reports and expression systems, suggesting a regulatory mechanism that has yet to be described^5–9^.

We have recently reported a detailed description of the cell-to-cell variability of Kv1.2 regulation, by characterizing its property of ‘use-dependent activation’^5, 10^. This describes potentiation of Kv1.2 channel activity in response to prior stimuli (either long depolarizing prepulses, or repetitive trains of brief depolarizations), and is a reflection of rapid switching of the channel between gating modes with different voltage sensitivity. We have observed this behavior in Kv1.2 currents recorded in heterologous mammalian cell lines and in primary neuronal cultures, and its marked cell-to-cell variability has been interpreted to suggest the involvement of an extrinsic mechanism^5, 6, 10^. Use-dependent activation can be abolished by various mutations of Thr252 in the S2-S3 linker. However, it has remained unclear what cellular variables promote occupancy of the diverse gating modes of Kv1.2.

In comparison to the inner workings of voltage sensitivity, regulation of ion channels by extrinsic regulators has received less attention, although auxiliary protein and lipid regulators clearly have important functional and physiological effects^11–13^. The most widely recognized auxiliary subunits of Kv1.2 are the family of Kvβ subunits^11, 14, 15^, although other proteins (such as PSD-95, cortactin, RhoA) and lipids have been suggested to interact and regulate expression and/or gating of Kv1.2^16–19^. The importance of understanding regulatory mechanisms is highlighted by the recognition that heteromeric assembly of ion channel subunits often enables recruitment of sensitivity to diverse signaling pathways^20, 21^. This is also true of Kv1.2, which assembles in heterotetrameric complexes with other Kv1 channels, and can recruit sensitivity to use-dependent activation^5^.

In this study, we report the surprising finding that use-dependent activation of Kv1.2 is regulated by the redox environment. Exposure of Kv1.2 to reducing conditions causes these channels to exhibit pronounced use-dependent activation as channels ‘escape’ from the inhibited gating mode upon membrane depolarization. Channels can be shuttled on the time scale of seconds between an inhibited gating mode (favored by reducing agents) and a potentiated gating mode (populated after strong or repetitive depolarizations). Using membrane-impermeant reducing agents (tris(2-carboxyethyl)phosphine (TCEP), glutathione (GSH) and cysteine (Cys)), we demonstrate that this effect is exclusively controlled by the extracellular redox potential, and can be recruited to heteromeric Kv1 channels with one or more Kv1.2 subunits. Overall, we demonstrate a novel mechanism of regulation of Kv1.2 channel complexes by the extracellular redox potential.

## Results

### Redox conditions strongly regulate voltage-dependence of Kv1.2

**Figure 1:**
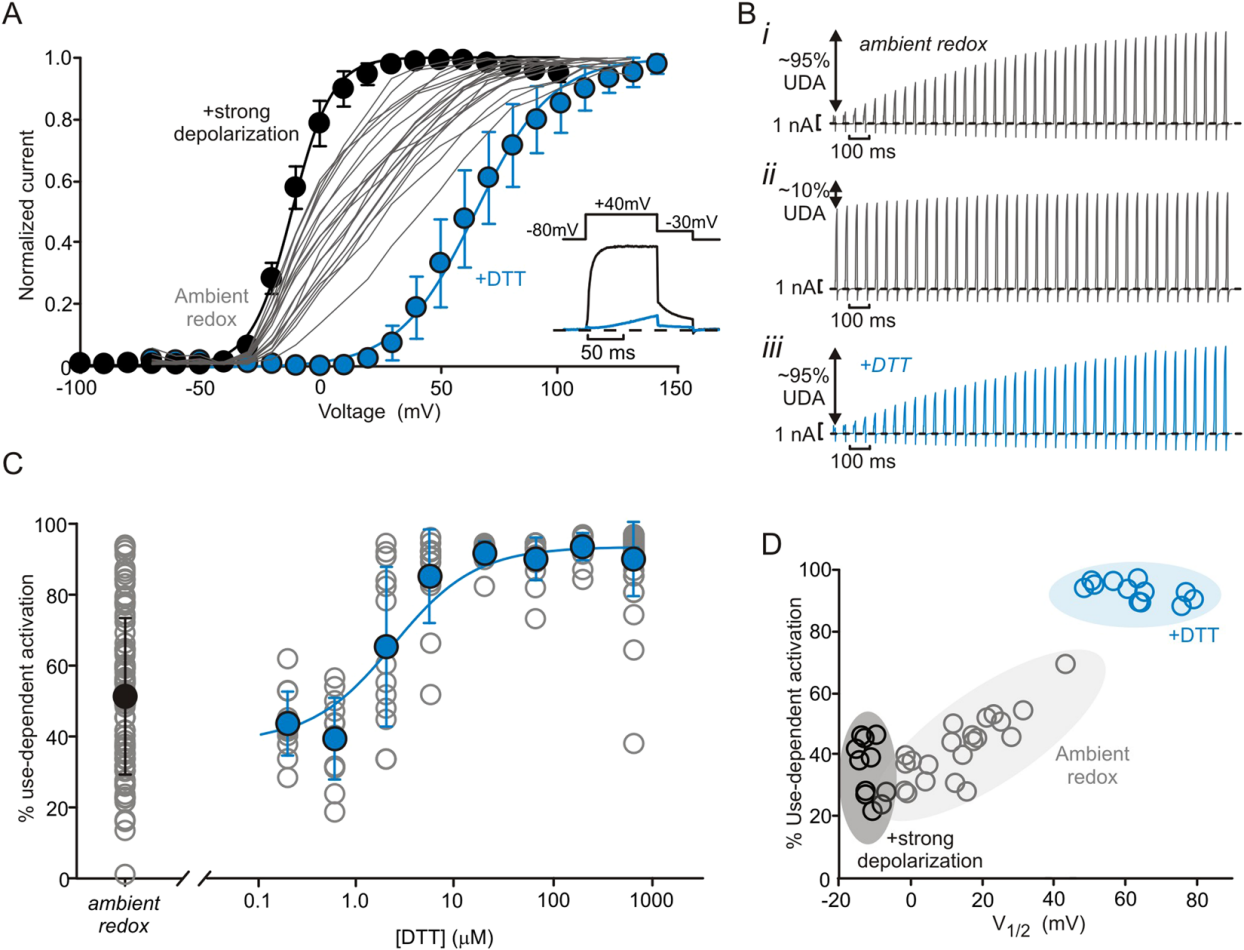
Reducing conditions promote use-dependent activation. Conductance-voltage relationships were recorded from tail current amplitudes at −30 mV (see inset) in ltk- fibroblast cells expressing Kv1.2. Grey lines are conductance-voltage relationships from multiple individual cells in ambient redox conditions (spread of V_1/2_ is from −1.7 to +43 mV). Mean conductance-voltage relationships (±S.D.) are shown for cells incubated in 666 µM DTT (N = 13, blue symbols, V_1/2_ = +64 ± 11 mV), or collected with a modified protocol that delivers a 500 ms depolarization to +60 mV before each voltage sweep (black symbols, N = 11, V_1/2_ = −11 ± 3 mV). (Inset) Currents elicited with a + 40 mV depolarization illustrate the suppression of current in DTT. (**B**,**i**–**iii**) Kv1.2 expressing cells were stimulated with repetitive 10 ms depolarizations from a holding potential of −80 mV to + 60 mV (20 Hz frequency). Different sample sweeps reflect the variability of use-dependence in ambient redox (i,ii), and a shift towards strong use-dependence after incubation in DTT (iii). (**C**) % use-dependent activation (UDA) is calculated as the fraction of activating current during the pulse train: (pulse 100 peak current – pulse 1 peak current)/(pulse 100 peak current), (blue symbols: mean ± S.D., grey symbols: data for individual cells in each condition; N = 87 for ambient redox, N = 44 for 666 µM DTT, and N = 12–14 for other DTT concentrations). DTT dependence of average UDA was fit with a Hill equation (EC50 = 1.6 µM and a Hill coefficient of 1.0). (**D**) Cell-by-cell correlation between % use-dependent activation and V_1/2_ measured in ambient redox (gray, N = 21), DTT (blue, N = 13), or after potentiation by strong prepulses (black, N = 11).

Despite being a member of the well-characterized *Shaker*-related Kv1 family, and described structurally at atomic resolution^4, 22^, there is poor understanding of the dramatic variability of Kv1.2 channel gating when expressed in mammalian cell lines and neurons^5^. Under ambient redox conditions, we observed that cells expressing Kv1.2 channels exhibit a wide range of V_1/2_ of activation, from −1.7 mV to +43 mV, illustrated by data collected from individual cells (Fig. 1A, gray lines). This variability reflects a distribution between two extreme gating modes that can been favored by either strong depolarizing prepulses^5, 6^, or, as we show here, redox environment. In the presence of dithiothreitol (DTT) (Fig. 1A, blue), channel activation clusters towards a strongly depolarized V_1/2_ of +64 ± 11 mV – we describe these channels as operating in an ‘inhibited’ gating mode that resists opening. In contrast, exposure of channels to a strong depolarization prior to measuring channel activation normalizes the conductance-voltage relationship to a much more hyperpolarized V_1/2_ of −11 ± 3 mV, reflecting the voltage-dependence of activation of channels in a ‘potentiated’ gating mode (Fig. 1A, black). Additionally, there is a change in the steepness of the activation curve reflected in a slope factor (k) value of 16.5 ± 3.0 mV for DTT-treated cells and 8.5 ± 1.6 mV for cells exposed to a strong depolarization. These data demonstrate a much larger dynamic range of activation properties of Kv1.2 than has been previously recognized, with a difference of ~75 mV between the extreme gating modes. These findings also illustrate how channels can be manipulated to gate nearly uniformly in either mode, using strong depolarizing prepulses (potentiated mode) or reducing conditions (inhibited mode).

The bi-modal gating of Kv1.2 gives rise to a unique regulatory property that we have described as use-dependent activation, characterized by pronounced current potentiation during trains of repetitive depolarizations (Fig. 1B,i). Upon depolarization, channels initially in the inhibited mode will resist opening, but upon opening they appear to switch into the potentiated mode. In this way, channels slowly accumulate in the potentiated mode that is permissive to opening leading to a progressive pulse-by-pulse increase in current^10^. We assessed the variability of Kv1.2 use-dependent activation by delivering trains of 10 ms depolarizations (+60 mV, 20 Hz), and measuring the percent difference between the current elicited by the first and last pulse of the train (‘% use-dependent activation’). Under ambient redox conditions, some cells exhibit a dramatic increase in current (Fig. 1B,i), whereas other cells exhibit negligible use-dependence (Fig. 1B,ii). However, in the presence of the reducing agent DTT, cells are shifted quite uniformly to a pronounced use-dependent phenotype (Fig. 1B,iii,C). In a concentration-response experiment, 10–30 μM DTT was sufficient to strongly bias use-dependent behavior (EC50 of 1.6 μM DTT, Fig. 1C). Note that the use-dependent activation phenotype is a functional reflection of the bi-modal gating properties of Kv1.2, and is a good surrogate measure for V_1/2_ of channel activation (Fig. 1D). Shown on a cell-by-cell basis, V_1/2_ correlates with the extent of use-dependent activation in ambient redox (Fig. 1D, grey circles), and both phenotypes are shifted together upon exposure to DTT (Fig. 1D, blue circles). We also tested the effects of a variety of redox species on use-dependent activation (Supplementary Fig. 1). In the presence of oxidizing agents, use-dependent activation is not abolished, but rather retains the substantial variability observed in ambient redox conditions.

### DTT accelerates recovery of use-dependent gating

**Figure 2:**
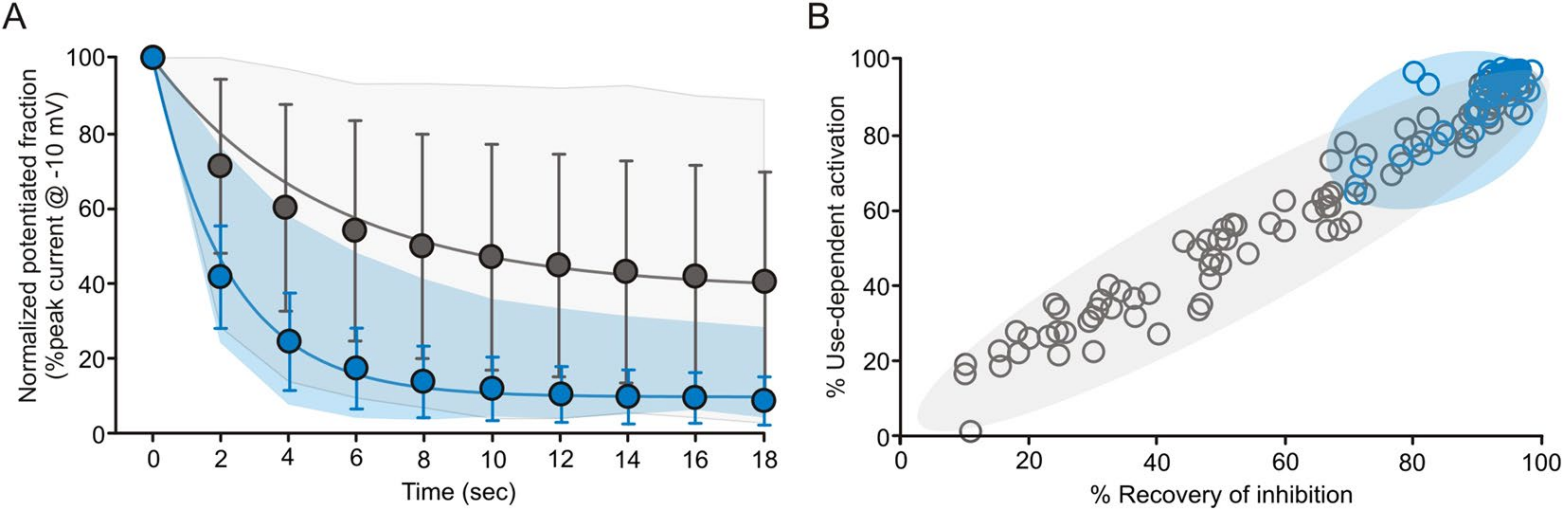
Time dependent recovery of inhibited gating mode. (**A**) Cells expressing Kv1.2 were stimulated with a train of repetitive depolarizations to +60 mV, followed by a sequence of 15 ms pulses to −10 mV (0.5 Hz) to assess recovery of the inhibited gating mode (detailed protocol in Supplemental Figure 2). Mean normalized test pulse magnitude (±S.D) for ambient redox (grey, time constant = 5.1 ± 2.9 s, N = 51) and DTT incubation (blue, time constant = 2.2 ± 1.0 s, N = 46) are presented, along with surfaces illustrating the range of variability observed in individual cells. (**B**) Cell-by-cell correlation of % use-dependent activation and % recovery of inhibition. Each data point reflects an individual cell (grey: ambient redox, blue: 666 µM DTT).

Use-dependent activation is reversible within seconds if channels are held closed with hyperpolarization, as potentiated channels can spontaneously revert to the inhibited gating mode. To quantify the rate and extent of recovery, we first potentiated Kv1.2 channels with repetitive depolarizations, then returned the voltage to −80 mV and delivered 15 ms depolarizations to −10 mV every 2 s (Supplementary Fig. 2A). At −10 mV, only channels in the potentiated gating mode can open significantly (see activation curves in Fig. 1A). Immediately after the depolarizing train, most channels are potentiated, yielding large currents at −10 mV. Channels then gradually revert to the inhibited gating mode, causing test pulse currents to decline (Fig. 2A). The rate of recovery was highly variable in ambient redox conditions, illustrated by the grey area delimiting the extreme boundaries of recovery kinetics in ambient redox (data from individual cells are presented in Supplementary Fig. 2B,C). To minimize variability and avoid complications arising from oxidation of DTT in solutions, we used a high concentration of DTT to test recovery in reducing conditions. The rate of return of current inhibition in 666 µM DTT was nearly 2.5-fold faster and more uniform (τ = 2.2 ± 1.0 s in DTT vs 5.1 ± 2.9 s in ambient redox, Fig. 2A, see also Supplementary Fig. 2). We measured the % recovery of inhibition as the % difference between the first and final recovery test pulses. On a cell-by-cell basis, there was a close correlation between the extent of use-dependent activation and recovery of inhibition (Fig. 2B, gray circles). This illustrates that cells recover robustly to the level of use-dependence observed initially after whole cell break-in. After exposure to 666 μM DTT, recovery of use-dependence is accentuated, with cells predominantly exhibiting pronounced use-dependent activation and rapid complete recovery of inhibition (Fig. 2B, blue circles). These findings reinforce that use-dependent activation reflects a reversible shift between inhibited and potentiated gating modes, and is strongly influenced by redox conditions.

### Kv1.2 recruits redox sensitivity to heteromeric channel complexes

**Figure 3:**
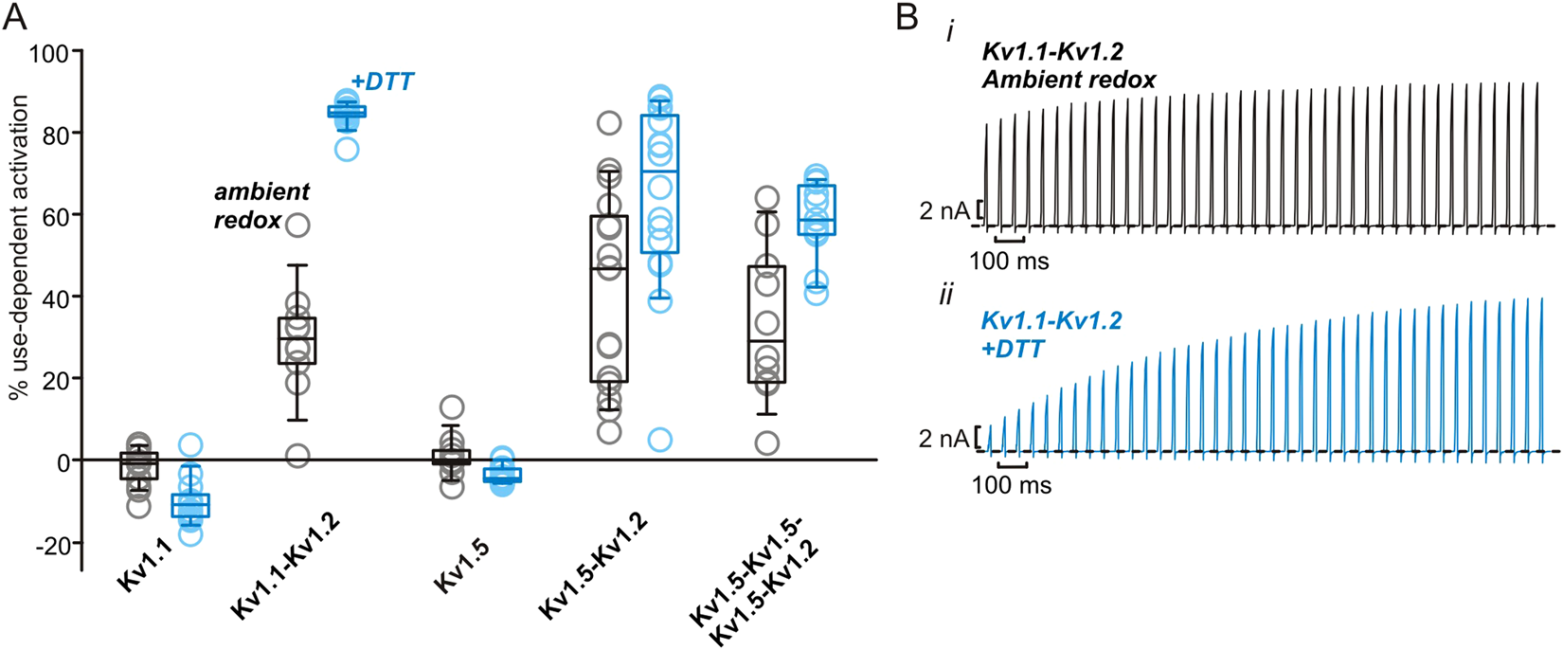
Redox-sensitive use-dependence is transferrable in heteromeric channels containing Kv1.2 subunits. (**A**) Repetitive depolarizations were used to assess % use-dependent activation, as described in Fig. 1B, for a variety of Kv1 channel subtypes and concatenated combinations with Kv1.2 as indicated. Each data point reflects the % use-dependence recorded from an individual cell in ambient redox conditions (gray) or in the presence of either 666 µM or 2 mM DTT (blue). N = 10–17 for each construct and condition. (**B**) Exemplar current tracings illustrating use-dependent activation of Kv1.1-Kv1.2 dimeric channels in (i) ambient redox conditions or (ii) 666 µM DTT.

Kv1.2 channels frequently assemble with other Kv1 subunits to generate heteromeric channels. However, only Kv1.2 has been reported to exhibit use-dependent activation among the Kv1 family, and all but Kv1.2 possess a Lys or Arg at the critical Thr252 position previously shown to be essential for the Kv1.2 gating mode shift^6^. We have previously reported robust use-dependent activation in Kv1.2-containing heteromeric complexes, with one or two Kv1.2 subunits being sufficient to impart use-dependence^5^. We tested redox sensitivity of use-dependence in other Kv1 channels and heteromeric complexes, as we were uncertain whether the accentuated use-dependent behavior in reducing conditions would evoke a latent use-dependent phenotype in closely related channels. Kv1.1 homomeric channels exhibit no apparent use-dependent activation in ambient redox conditions, or after incubation in up to 2 mM DTT (Fig. 3A and Supplemental Fig. 3). However, using concatenated Kv1.1-Kv1.2 heterodimeric constructs (1:1 Kv1.1:Kv1.2 stoichiometry), modest use-dependent activation was apparent in ambient redox, together with a profound shift to use-dependent behavior in reducing conditions (Fig. 3A,B). Kv1.2-Kv1.5 heterodimers showed some redox sensitive use-dependence, although not as prominent as WT Kv1.2. Additionally Kv1.2-1.5-1.5-1.5 heterotetrameric constructs (1:3 Kv1.2:Kv1.5 stoichiometry) responded sub-maximally to 2 mM DTT (Fig. 3A). These findings illustrate that Kv1.2 can act as a module that recruits use-dependent activation and strong redox sensitivity to heteromeric Kv1 channel complexes, although the extent of these effects may depend on subunit composition.

### Extracellular redox environment controls Kv1.2 channel gating

**Figure 4:**
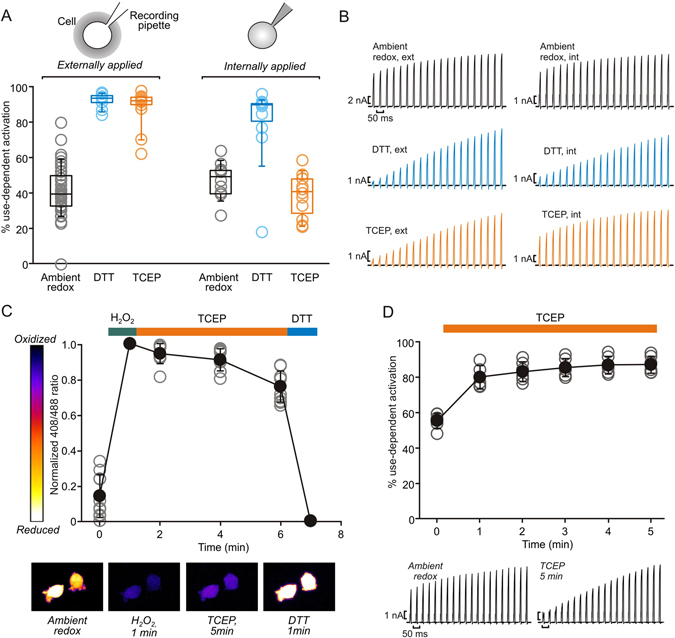
Extracellular redox environment modulates Kv1.2 use-dependent activation. (**A**) Use-dependent activation was assessed using repetitive depolarizations as described in Fig. 1. DTT (membrane permeant, blue) or TCEP (membrane impermeant, orange) reducing agents were applied intracellularly at 500 µM (through the pipette solution) or extracellularly at 200 µM, as indicated (External agents: N = 41 for ambient redox, N = 12 for DTT and N = 15 for TCEP; internal agents: N = 12 for ambient redox, DTT and TCEP). (**B**) Exemplar current traces from cells expressing Kv1.2 channels in various reducing agents illustrate the membrane sidedness of TCEP effects. (**C**) Mouse ltk- fibroblasts transfected with the genetically encoded redox sensor Grx1-roGFP were perfused with a range of redox active compounds, as indicated (H_2_O_2_ at 1 mM, DTT and TCEP at 666 µM). Extracellular TCEP provides minimal recovery of intracellular redox potential, while DTT rapidly reduces the intracellular compartment (black symbols = mean ± S.D., grey symbols are data from individual cells, N = 10). Pseudo-colouring was generated by the ‘Fire’ lookup table in ImageJ. (**D**) Use-dependent activation was assessed under ambient redox conditions (time = 0) with repetitive depolarizations as described in Fig. 1B, followed by perfusion with 200 µM extracellular TCEP (N = 6). TCEP produces a maximal shift in use-dependent activation within 1–2 min, also illustrated with exemplar current traces in lower panels.

Since DTT is membrane permeable, it does not reveal the sidedness of redox sensitivity. DTT application either extracellularly (in the external solution) or intracellularly (in the internal pipette solution) is sufficient to accentuate use-dependent activation (Fig. 4A and B, blue). The membrane impermeable reducing agent (TCEP) also potentiated use-dependent activation, much like DTT, when applied extracellularly. In contrast, internally applied TCEP failed to promote use-dependent activation (Fig. 4A and B, orange). Other extracellularly applied membrane impermeable reducing agents (GSH and Cys) mimicked the effects of extracellular TCEP and DTT. We measured the concentration-response of use-dependence to each of these membrane impermeable reducing agents and found that each had an EC50 of ~2–3 μM (Supplementary Fig. 4).

To confirm that TCEP was exclusively controlling Kv1.2 via the extracellular compartment, we tracked the intracellular redox potential using a genetically encoded ratiometric redox sensor, Grx1-roGFP. In ambient redox conditions, the intracellular redox potential is strongly reducing, reflected by a low 408/488 ratio (Fig. 4C). Perfusion with 1 mM H_2_O_2_ (membrane permeable) quickly leads to oxidation of the redox potential, reflected in an increased 408/488 ratio (see methods for ratio calculation). Subsequent addition of extracellular TCEP (666 µM) for up to 5 min did not markedly restore the intracellular redox potential, while addition of DTT (666 µM) rapidly reduced the intracellular redox potential. These slow/absent effects of TCEP on the intracellular redox potential contrast with the time course of extracellular TCEP effects on Kv1.2 gating, which are nearly complete within one minute of exposure to 200 µM TCEP (Fig. 3D). These findings confirm that the gating effects described in Figs 1, 2 and 3 are strongly controlled by changes to the extracellular redox potential.

### Cysteine residues in Kv1.2 do not control redox sensitivity

**Figure 5:**
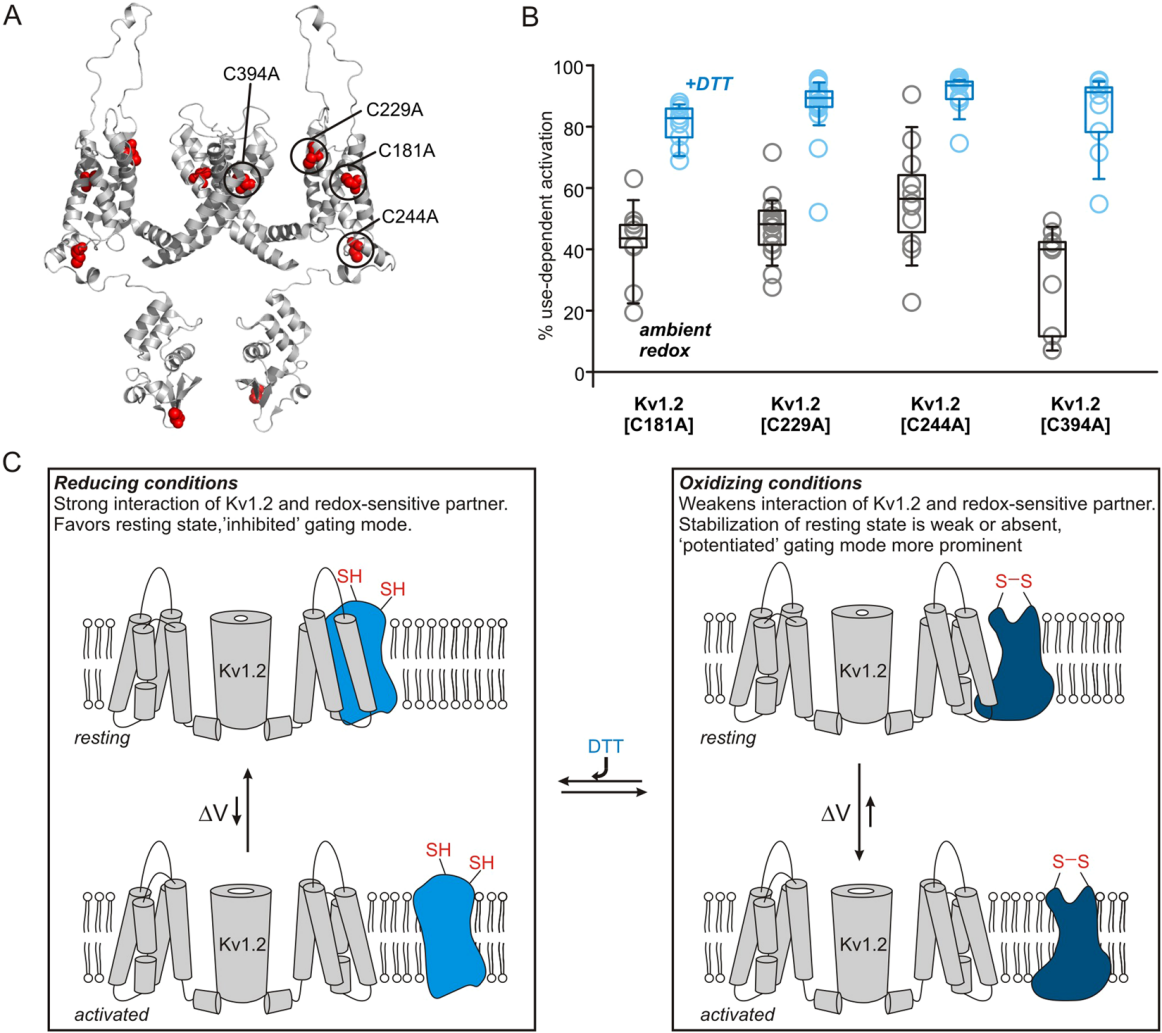
Systematic mutagenesis of transmembrane cysteine residues in Kv1.2. (**A**) Cysteine residues are highlighted in red on the Kv1.2 structure (PDB 3LUT). C181, C229, C244, and C394 in the transmembrane domains were mutated to alanine. (**B**) Use-dependent activation of each cysteine mutant was assessed with trains of repetitive depolarizations, as described in Fig. 1, in ambient redox and after incubation in 666 µM DTT (N = 10–22 for each condition). (**C**) Schematic model depicting redox dependent interactions of Kv1.2 with a postulated extrinsic binding partner (blue) that mediates use-dependent activation. We propose that the reduced state of the regulatory partner has a high affinity for the Kv1.2 channel that promotes an inhibited gating mode by stabilizing the resting conformation. In more oxidizing conditions, the regulatory partner has weakened or altered interactions with Kv1.2, causing the inhibited gating mode to be less prominent.

Use-dependent activation of Kv1.2 has been reported to show significant cell type variation. It is apparent in all mammalian cell lines we have tested to date, but is not reported when Kv1.2 is expressed in *Xenopus laevis* oocytes, suggesting it may not be an intrinsic property of the channel^6, 9, 23, 24^. To investigate potential determinants of redox sensitivity in Kv1.2, we systematically mutated all cysteines within the channel transmembrane domains to alanine (Fig. 5A). None of these mutations abolished use-dependent activation properties (Fig. 5B, black), and all Cys → Ala mutants retained sensitivity to reducing agents (Fig. 5A, blue). Based on these findings, the redox sensitivity of Kv1.2 does not appear to arise from modification or formation of a disulfide involving a cysteine that is native to the channel. We have previously suggested that an extrinsic inhibitory regulatory protein or molecule interacts with Kv1.2 to generate use-dependent activation, with variable stoichiometric abundance of this regulatory partner leading to variable degrees of use-dependent activation under ambient conditions^10^. Our current findings seem consistent with this hypothesis, and lead to a further suggestion that the extrinsic regulator (or its interaction with the channel) is sensitive to extracellular redox conditions. In our model, Kv1.2 is depicted as having a high affinity for the inhibitory regulator in its reduced state (perhaps when a key disulfide is broken), leading to stabilization of the channel resting state, and a weaker interaction with the regulator in its oxidized state (Fig. 5C). The interaction between the regulator and the channel can also be weakened by depolarization leading to channel opening. Based on our observations that oxidizing agents do not alter use-dependent activation beyond the ambient redox condition (Supplementary Fig. 1), we would predict that the oxidized state of the regulator has a weakened (but non-zero) affinity for the channel.

### Calibrated redox potential measurements of Kv1.2 use-dependent gating

**Figure 6:**
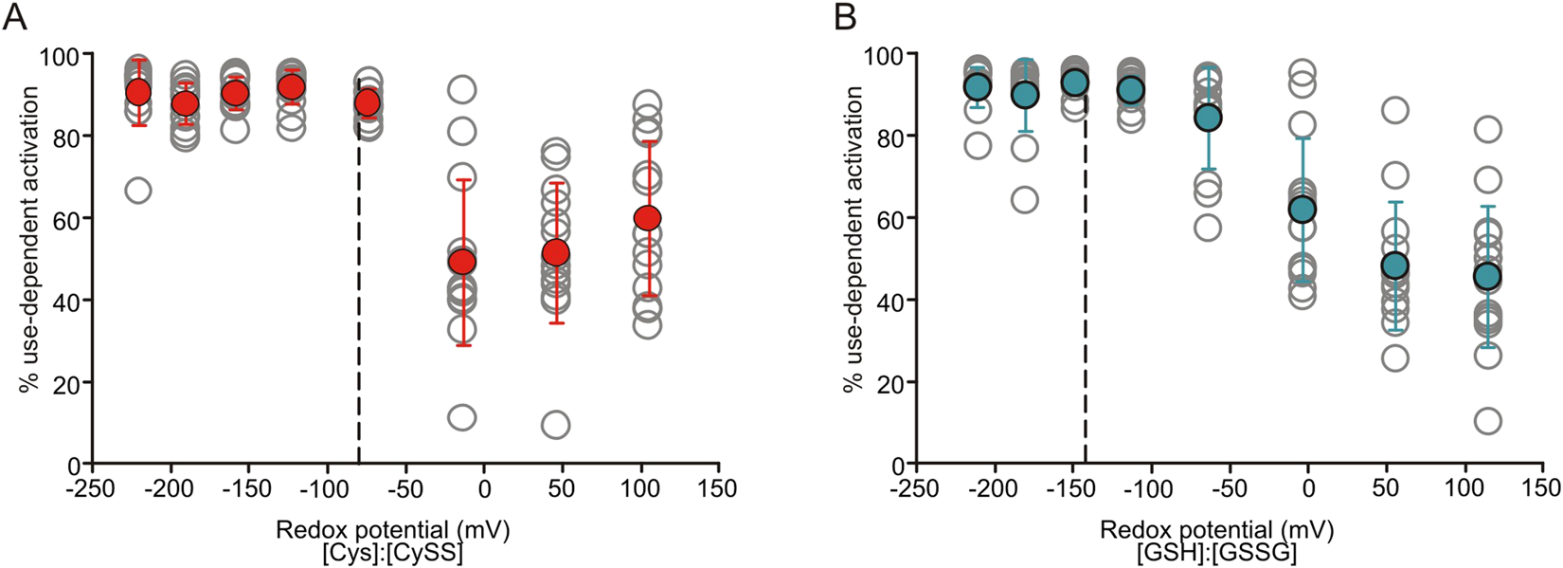
Calibrated effects of redox potential on Kv1.2 use-dependent activation. (**A**,**B**) Extracellular redox potential was clamped using membrane impermeant redox couples ((**A**), cysteine and cystine (Cys:CySS), or (**B**), reduced and oxidized glutathione (GSH:GSSG)) by varying the ratio of the two species while maintaining a total concentration of 100 µM. N = 13–15 for each redox potential.

Using membrane impermeant redox couples, we clamped the extracellular redox potential to calibrate the redox sensitivity of the use-dependent effect. We measured use-dependence after incubation in a range of cysteine:cystine (Cys:CySS) ratios, the most prominent physiological extracellular redox couple (Fig. 6A). At more positive redox potentials (ie. more oxidized conditions, low Cys:CySS ratio), there was wide cell-to-cell variability of use-dependent activation. At negative redox potentials (more reducing conditions, high Cys:CySS ratio), use-dependent activation became far more pronounced between −50 mV and −100 mV. The approximate redox potential of the Cys:CySS couple in plasma (−80 mV) is plotted as a dashed line for reference, suggesting that use-dependent activation is sensitive to extracellular redox potentials in this range, but distant from typical cytoplasmic redox potentials (~−270 mV)^25, 26^. We performed a similar set of calibrating experiments using the reduced glutathione:oxidized glutathione (GSH:GSSG) ratio to clamp the extracellular redox potential, and observed that use-dependent activation was also strongly shifted in the range of 0 mV to −100 mV (Figure 6B), similar to the Cys:CySS redox buffer.

## Discussion

Despite evolving into an archetype of voltage-dependent gating, and providing the rare luxury of directly comparing ion channel function with atomic resolution structure, there is a lack of understanding of regulatory mechanisms that generate considerable variability in the voltage-dependent activation of Kv1.2. There is marked inter-report variation in the parameters of voltage-dependent activation of Kv1.2, including significant dependence on expression system^6, 9, 13^. Moreover, other reports have described significant ‘intra-experimental’ or ‘pulse-to-pulse’ variation in Kv1.2 that has been challenging to explain^6, 7^. These past studies clarified that depolarizing voltages could reversibly populate Kv1.2 channels in a potentiated/facilitated mode, although it remained unclear what signals governed the inhibited gating mode. We report here that occupancy of the inhibited gating mode is strongly favored by mild reducing conditions in the extracellular compartment. The dramatic effect of reducing conditions has allowed us to demonstrate that Kv1.2 channel activation can be modulated reversibly over a far greater range of voltages (between V_1/2_ of −11 mV and +64 mV) than was previously recognized. This basic observation will hopefully pave the way to unraveling molecular details of this poorly understood regulatory mechanism of Kv1.2.

Beyond its importance as a biophysical model of ion channel gating, Kv1.2 appears to be an essential contributor to neurological function in humans and animal models. Severe effects of Kv1.2 deletion in mice demonstrates that loss-of-function of Kv1.2 is very poorly tolerated compared to knockout of other Kv1 channel subtypes, as Kv1.2-deficient mice die within weeks of birth due to severe generalized seizures^27–29^. A growing number of reports have linked Kv1.2 mutations to neurological diseases^30, 31^. Mutations causing either gain- or loss-of-function phenotypes of Kv1.2 have been linked to epileptic encephalopathy and ataxia^27, 32, 33^, and there is a notable low frequency of predicted loss-of-function Kv1.2 mutants (along with Kv1.4) in exome aggregation databases (ExAC consortium)^34^. It should be recognized that a role for use-dependent activation of Kv1.2 has not yet been tested in these mutants when exposed to physiological extracellular redox potential. However, use-dependent activation is a unique property of Kv1.2 among the Kv1 family, we have also demonstrated previously that use-dependent activation can be detected in toxin-subtracted Kv1.2 currents recorded in primary neuronal cultures, and the redox-dependence we have observed falls in the physiological range of extracellular redox potential^5, 25, 26^. Taken together, it is fair to say that Kv1.2 appears to be critical for normal neurological function and other Kv1 channels cannot compensate for its loss.

‘Moment-to-moment’ variation of Kv1.2 activation properties is hypothesized to reflect channel regulation by an extrinsic binding partner that stabilizes the resting channel conformation, and thereby causes channels to exhibit the ‘inhibited’ gating mode^5, 10^. We propose that this binding partner is sensitive to extracellular redox conditions, which influence its interactions with Kv1.2 channels. Several experimental observations suggest this is a reasonable mechanism. Firstly, there is considerable cell-to-cell variability under ambient redox conditions (even when redox is buffered at mild oxidized potentials, Fig. 6 and Supplemental Fig. 1), which may be explained by variable stoichiometric ratios of Kv1.2 and its regulatory partner(s)^6^. We speculate that in reducing conditions, the affinity of Kv1.2 interactions with its binding partner is sufficiently high that most/all channels are bound. However, in ambient/oxidizing conditions (weaker channel:regulator interaction), only cells with high expression of the putative binding partner will exhibit prominent use-dependent activation. A second critical observation is that mutation of all transmembrane cysteine residues in the channel (as potential intrinsic redox sensors) fails to abolish use-dependent activation or redox sensitivity (Fig. 5). We have not exhaustively tested other possible redox sensitive side chains such as methionine, although it is noteworthy that concentrations of DTT reported to be required for reduction of methionine sulfoxide are far greater than the mild DTT concentrations (and short durations) that appear to be sufficient for a dramatic gating effect in Kv1.2 (Figs 1 and 4)^35^. Also, a redox sensor intrinsic to the channel does not readily explain the large cell-to-cell variability of this phenomenon. Thirdly, expression of Kv1.2 in certain common expression systems like *Xenopus laevis* oocytes fails to reconstitute use-dependent activation or redox sensitivity, suggesting that these properties are not intrinsic to the channel^23, 24^. Lastly, it is intriguing that the only mutations identified thus far that can attenuate use-dependent activation (one of which is Kv1.2 residue T252) lie at the intracellular side of the channel (on the S2–3 linker), while the gating effect is clearly modulated by an extracellular signal. Due to this apparent transmembrane cross-talk, our ongoing investigation of use-dependence is primarily focused on the identification of interacting proteins, although we do not have absolute evidence to rule out the possibility that a lipid or other class of signaling molecule is involved.

Other ion channel types have also been reported to be sensitive to the external redox environment, although this has generally been ascribed to intrinsic redox-sensing mechanisms. TRPC5 channels contain a disulfide in the S5–6 linker which is sensitive to extracellularly applied thioredoxin (Trx)^36^. Similarly, Cav3.2 channels are modulated by extracellular Trx, although in this case the extracellular redox sensor remains unidentified^37^. The NMDA (NR1/NR2A) receptor^38^, Orai1^39^, and ASIC channels^40^ all have intrinsic redox-sensitive elements that alter channel function. Perhaps the mechanism of extracellular redox sensitivity that is most analogous to the mechanism we have proposed is the regulation of β3 subunit effects on BK channels by extracellular redox. This appears to be related to the formation of disulfides in the extracellular loop of β3^41^, thus extracellular redox sensitivity is imparted by disulfide formation/breakage on an auxiliary binding partner, similar to our proposed model (Fig. 5C), albeit with different functional outcomes. While the physiological roles of redox sensitivity of ion channels including Kv1.2 remain unclear, it is apparent that multiple channel types have evolved diverse mechanisms of redox responsiveness. We now have tools in hand to continue to investigate physiological roles of use-dependent activation of Kv1.2 and its regulation by redox. The importance of Kv1.2 for normal neurological function in humans and mice, together with the transferability of Kv1.2 use-dependent activation into heteromeric Kv1 complexes, suggests potentially important roles for this incompletely understood mode of regulation.

In summary, we have demonstrated redox conditions and patterns of voltage stimulation that bias homo- and heterotetrameric Kv1.2 channels into previously unrecognized extreme modes of channel gating. This variability of Kv1.2 gating relative to its close Kv1 family relatives underlies its unique but variable use-dependent activation properties. Ongoing investigation of the molecular basis for this process will hopefully reveal previously unrecognized binding partners and signaling mechanisms that control ion channel function.

## Methods

### Ion channel constructs and expression in cell lines

Kv1 channel cDNAs and dimeric constructs were expressed using the pcDNA3.1(-) vector (Invitrogen). For dimer construction, the leading protomer was subcloned into the NheI and XhoI sites in the pcDNA3.1(-) MCS. The trailing protomer was subcloned into the EcoRI and HindIII sites. Monomeric constructs were subcloned into the EcoRI and HindIII sites. Constructs were all verified by diagnostic restriction digestions and Sanger sequencing (Genewiz, Inc.).

Mouse ltk- fibroblast cells were maintained in culture in a 5% CO_2_ incubator at 37 °C in DMEM supplemented with 10% FBS and 1% penicillin/streptomycin. Cells were split onto sterile glass coverslips and, 12 hrs later, were transfected with channel cDNAs using jetPRIME transfection reagent (Polyplus). Cells were co-transfected with fluorescent proteins to allow identification of cells for recording by epifluorescence. Recordings were done 24–48 hr following transfection.

### Electrophysiology

Patch pipettes were manufactured from soda lime capillary glass (Fisher), using a Sutter P-97 puller (Sutter Instrument). When filled with standard recording solutions, pipettes had a tip resistance of 1–3 MΩ. Recordings were filtered at 5 kHz, sampled at 10 kHz, with manual capacitance compensation and series resistance compensation between 70–90%, and stored directly on a computer hard drive using Clampex 10 software (Molecular Devices). Bath solution had the following composition: 135 mM NaCl, 5 mM KCl, 1 mM CaCl_2_, 1 mM MgCl_2_, 10 mM HEPES, and was adjusted to pH 7.4 with NaOH. Pipette solution had the following composition: 135 mM KCl, 5 mM K-EGTA, 10 mM HEPES and was adjusted to pH 7.2 using KOH. Chemicals were purchased from Sigma-Aldrich or Fisher.

Unless otherwise indicated, recordings in reducing conditions were carried out by incubating cells in the indicated reducing agent (diluted in serum-free DMEM media) for 5–20 min prior to recording. During the recordings, cells were continuously bathed in extracellular solution with the indicated reducing agent. All reducing agents were stored as stock solutions at −20 °C and diluted just prior to experimental use to minimize spontaneous oxidation. For intracellular application of reducing agents, cells were held for five minutes after whole-cell break-in before recording, to allow equilibration with the pipette solution.

### Grx1-roGFP

To track the intracellular redox potential of mouse Itk- fibroblast cells, we transiently transfected the pLPCX cyto Grx1-roGFP2^42^ construct using jetPRIME transfection reagent. After 24 hours, cells were placed in the imaging chamber with bath solution flowing. Fluorophores were excited with wavelengths of either 408 nm or 480 nm (Mightex LED light source, filtered; Mightex), and emission intensity at 510 nm was determined by capturing images (Hamamatsu ORCA Flash 2.8, HC Image software, Hamamatsu) and subsequent processing. Images were background subtracted, and the ratio of emission with 408 nm and 480 nm excitation was calculated on a cell by cell basis. Using ImageJ, the intensity within a region of interest drawn around one cell was subtracted from the background. This corrected intensity at the 408 nm excitation was divided by that at 488 nm excitation to calculate the 408/488 ratio. pLPCX cyto Grx1-roGFP2 was a gift from Tobias Dick (Addgene plasmid # 64975).

### Redox potential measurements

Two membrane impermeant redox couples were used to clamp the extracellular environment at different redox potentials: reduced/oxidized glutathione (GSH:GSSG) and cysteine/cystine (Cys:CySS). For the GSH:GSSG couple, different ratios of the oxidized and reduced forms were mixed to a final concentration of 100 µM to generate redox potentials between −210 mV and +115 mV. The redox potential was calculated using equation (1), where Eh is the redox potential, Eo is the standard reduction potential for glutathione (−240 mV), R is the gas constant, T is the temperature, z is the number of electrons transferred and F is Faraday’s constant.

1 $$Eh=Eo-\frac{RT}{zF}\,\mathrm{ln}(\frac{[GSH]}{{[GSSG]}^{2}})$$

The Cys:CySS couple was treated in a similar manner, with calculated redox potentials between −220 mV to +105 mV. The redox potential was calculated with equation (2), where the standard reduction potential for cysteine, Eo, is −250 mV.

2 $$Eh=Eo-\frac{RT}{zF}\,\mathrm{ln}(\frac{[Cys]}{{[CySS]}^{2}})$$

### Data analysis

Describing the variability of Kv1.2 gating is central to our findings, and so throughout the text we have displayed data for all individual cells collected, in addition to reporting mean ± SD or a box plot with the median. Dose-response curves to different reducing agents were generated using the Hill equation (3), where max and min UDA are the top and bottom of the dose-response curve and are fitted values, Kd is the dissociation constant, D is the concentration and n is the Hill coefficient.

3 $${\rm{ \% }}UDA=\frac{max\,UDA-\,min\,UDA}{(Kd/{D)}^{n}+1}+\,min\,UDA$$

Activation curves were fit with the Boltzmann equation (4), where I/Imax is the normalized current, V is the voltage applied, V_1/2_ is the half activation voltage, and k is a fitted value reflecting the steepness of the curve.

4 $$\frac{I}{Imax}=\frac{1}{1+{e}^{-(V-{V}_{1/2})/k}}$$

Activation curves from individual cells were fit, followed by statistical calculations for individual fit parameters. The time constant of recovery of inhibition was calculated by fitting an exponential decay equation (5) to the peak currents elicited by the −10 mV test pulse every 2 sec, where y is the % inhibited current at the given time (t), y_0_ is the total % inhibition in that cell, τ is the time constant of recovery of inhibition, and y_ss_ is the % current remaining after the cell returns to its maximal inhibition.

5 $$y={y}_{0}{e}^{-\tau /t}+{y}_{ss}$$

## Electronic supplementary material

Supplemental Information
