# Supplementary material for: Extracellular redox sensitivity of Kv1.2 potassium channels
Source: Sci Rep. 2017 Aug 22;7:9142. doi: 10.1038/s41598-017-08718-z (PMC5567313; doi:10.1038/s41598-017-08718-z)
Supplement: Supplementary file 1 — Supplemental Information [file 41598_2017_8718_MOESM1_ESM.pdf]

# Extracellular redox sensitivity of Kv1.2 potassium channels

Victoria A. Baronas, Runying Y. Yang, Harley T. Kurata

**SUPPLEMENTAL INFORMATION**

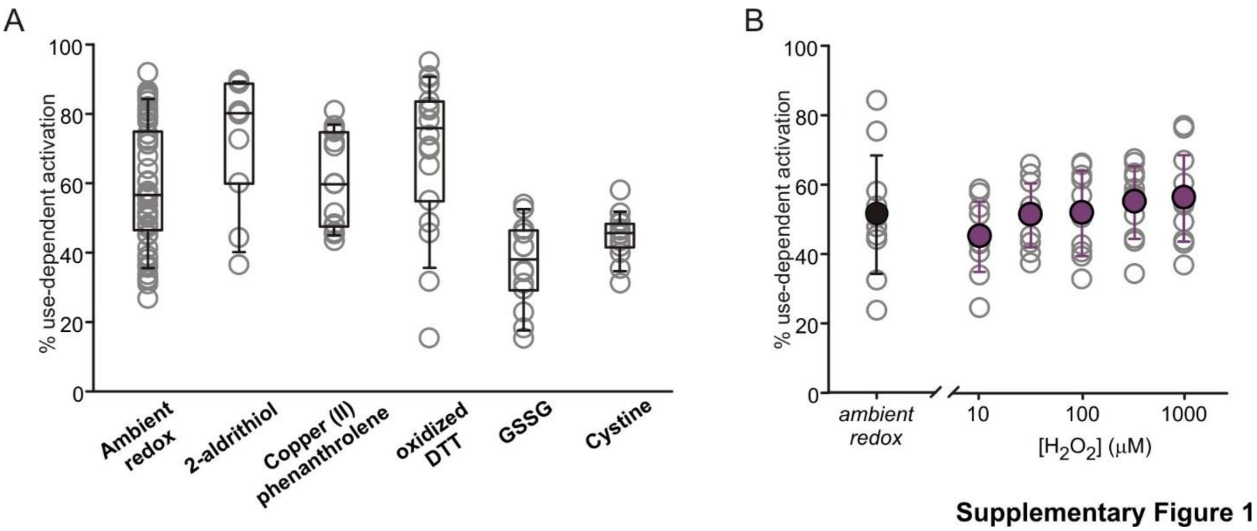


**Supplementary Figure 1**

**Supplemental Figure 1. Oxidizing agents do not affect use-dependent activation.** (A) Cells expressing Kv1.2 were incubated with various oxidizing agents, 2-aldrithiol (100 or 500 µM, N = 10), copper(II) phenanthrolene (2 µM/100 µM or 150 µM/500 µM, N = 14)), oxidized DTT (0.5 or 1 mM, N = 18), oxidized glutathione (GSSG) (1 mM, N = 14) and cystine (500 µM, N = 14), or in ambient redox (N = 57) and % use-dependence was calculated as described in Figure 1. (B) Use-dependent activation was also quantified in cells over a range of $H_2O_2$ concentrations (10 µM – 1 mM) with no effect observed.

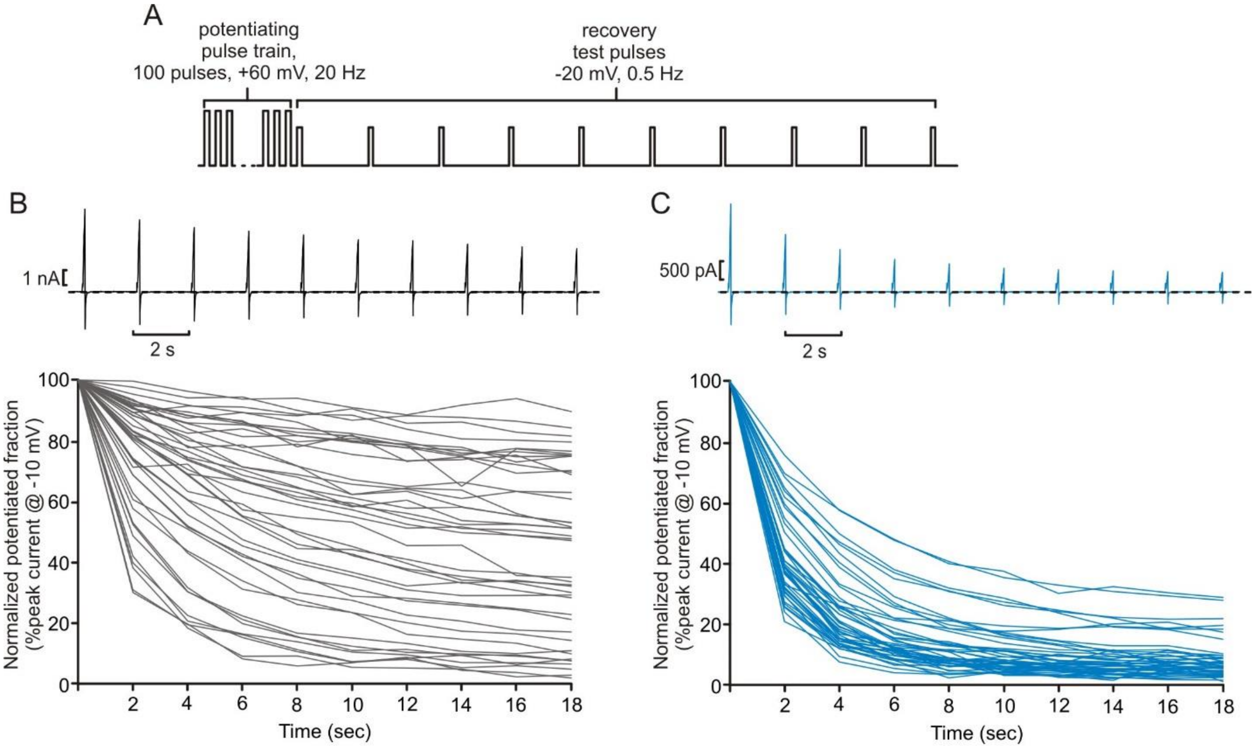


Supplementary Figure 2

**Supplemental Figure 2. Quantification of recovery of inhibited gating mode in ambient redox and reducing conditions.** (A) Detailed protocol for assessing recovery of inhibition. Cells were pulsed repetitively to +60 mV to populate the potentiated gating mode, then pulsed every 2 s to -10 mV to assess recovery to the inhibited gating mode. (B,C) Exemplar traces illustrate a sequence of recovery pulses in ambient redox (B) or 666 µM DTT (C). The time course of recovery is illustrated for all individual cells collected to illustrate the variability in ambient redox vs. DTT conditions.

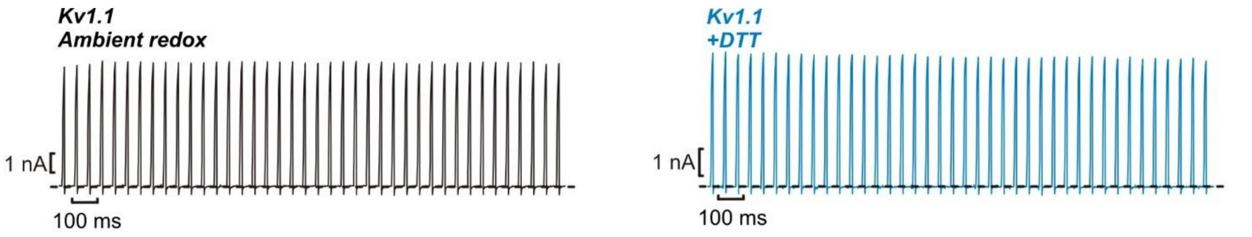


**Supplementary Figure 3**

**Supplemental Figure 3. Redox insensitive currents of Kv1.1 channels.** Exemplar currents recorded from ltk- mouse fibroblasts expressing Kv1.1 are presented in ambient redox conditions (black) or DTT (blue).

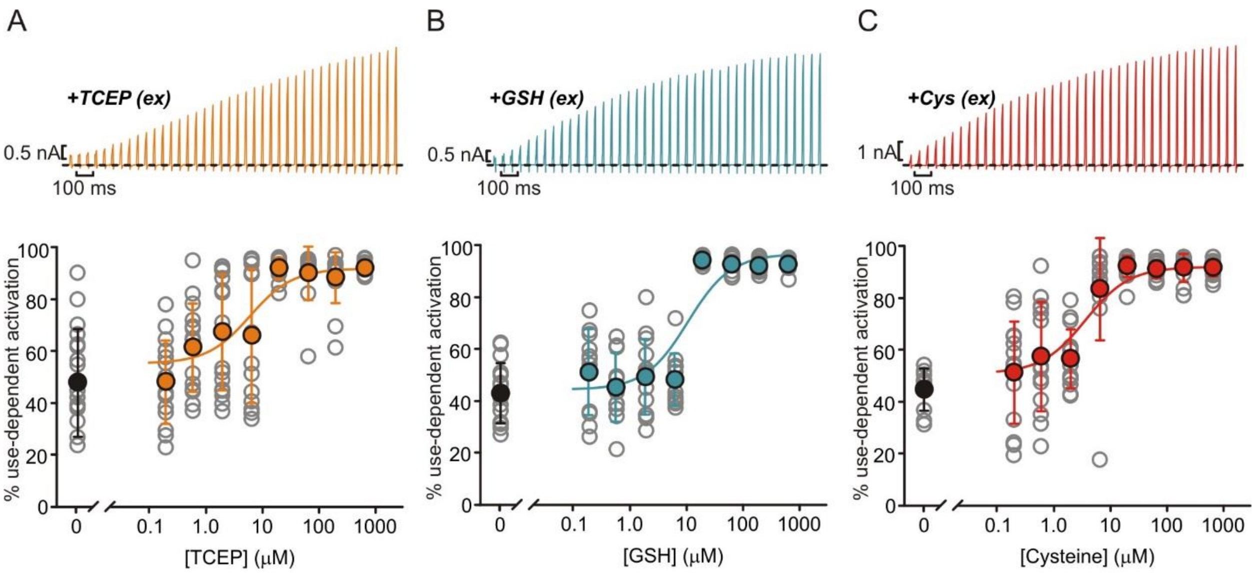


**Supplementary Figure 4**

**Supplemental Figure 4. Dose-dependent shift in use-dependence with membrane impermeant reducing agents.** (A-C) Concentration-response of use-dependent activation was measured with increasing concentrations of extracellularly applied (A) TCEP (EC50 of 3.4 μM and a Hill coefficient of 1.1), (B) reduced glutathione (GSH, EC50 of 2.1 μM and a Hill coefficient of 1.2), or (C) cysteine (EC50 of 2.8 μM and a Hill coefficient of 1.1). N = 10-20 for each condition.
